# Stable isotopes: their use and safety in human nutrition studies

**DOI:** 10.1038/s41430-020-0580-0

**Published:** 2020-02-11

**Authors:** Peter S. W. Davies

**Affiliations:** Child Health Research Centre Level 6, Centre for Children’s Health Centre, University of Queensland, 62 Graham Street, South Brisbane, QLD 4101 Australia

**Keywords:** Translational research, Obesity

## Abstract

Stable isotopes have been used as tracers in human nutritional studies for many years. A number of isotopes have been used frequently to assess body composition, energy expenditure, protein turnover and metabolic studies in general, such as deuterium (^2^Hydrogen), ^18^Oxygen, ^13^Carbon and ^15^Nitrogen. Nevertheless, there is still occasional confusion and concern over their safety, which can hinder the appropriate use of these isotopes in human studies. This mini review aims, therefore, to consider the safety of the four stable isotopes mentioned above, and to reiterate and reaffirm their safety once again. It is hoped that these data will be of use to new researchers in the field, as well as those considering the ethical or other implications of using these stable isotopes in nutritional research. Undoubtedly some of the confusion arises as deuterium, especially, is associated with the nuclear industry. However, as their name implies, of course, none of these stable isotopes are radioactive, and no adverse biological or physiological effects have been reported at the very low levels of enrichment that are used in human studies. There are ample data to reaffirm the safety of stable isotopes at the levels used in nutritional research, and unnecessary concerns and/or confusion should not be a block to continued use of these important tracers.

## Introduction

At the time of writing, it is 100 years since Francis Aston born in Birmingham in the United Kingdom, but then working at the renowned Cavendish Laboratory in Cambridge, described the evidence, at that time, that neon consisted of mainly two isotopes that were stable and were therefore not radioactive. This was not the first work with isotopes, but the majority of prior studies had been with some of the heavier elements, whilst Aston’s work was the first to suggest that isotopes existed amongst the naturally occurring main group of elements [1].

Stable isotopes are now known to exist in numerous elements with the key feature being that there are differing numbers of neutrons in the elements’ nucleus. Thus, one element can share the same atomic number, but differing mass numbers, which identifies the isotope. These differences do not alter to any great extent the chemical properties of the atom, but of course, do change the physical properties.

For example, the most common stable isotope of oxygen is ^16^Oxygen that is 99.76% of all oxygen found on the planet. The nucleus of this atom has eight protons and eight neutrons. A small amount of oxygen (0.04%) exists as ^17^Oxygen with eight protons but nine neutrons, and finally ^18^Oxygen (^18^O) constitutes 0.20% of all oxygen with again eight protons but this time ten neutrons. All three of these arrangements in the nucleus are, as the name implies, stable, and there is no radioactive decay or emission. Some examples of both stable and radioactive isotopes of some more common elements are shown in Table 1.

**Table 1:** Some examples of some stable, non-radioactive, isotopes used in nutritional research, and examples of some radioactive isotopes of the same elements.

Stable	Abundance	Radioactive	Half-life
^1^H	99.98%	^3^H	12.23 years
^2^H	0.02%		
^12^C	98.9%	^14^C	5730 years
^13^C	1.1%		
^14^N	99.6%	^13^N	9.9 min
^15^N	0.4%		
^16^O	99.76%	^14^O	70 s
^17^O	0.04%	^15^O	122 s
^18^O	0.20%		

Many of the stable isotopes shown in Table 1, have played an important role in nutrition research, and this mini review will focus on the application and safety of four often-used stable isotopes. They are not radioactive, and can be administered in small amounts in their role in nutritional research. They have long been a safe tool for assessing various aspects of human nutrition, and continue to be so, often in potentially vulnerable groups including, for example, premature infants, children in both health and disease and individuals with severe injuries [2–5]. Whilst, as previously stated, radioactivity is not an issue with these two isotopes, it is right and proper that other aspects of the safety of these stable isotopes be considered and understood prior to their use in human studies. The mechanism by which the use of stable isotopes may induce toxicity is via the so-called isotope effect. This effect stems from the fact that more energy, sometimes called activation energy, is required to split bonds between heavier isotopes, and this could lead to the slowdown of important physiological reactions, including enzyme activity. The magnitude of this potential effect is usually expressed as the ratio of the reaction rate of the lighter isotope to the heavier isotope [6]. Calculations show that by far the greatest isotope effect occurs with deuterium with a value of 18, whilst equivalent isotope effects for ^13^C, ^18^O and ^15^N are 1.25, 1.14 and 1.19, respectively [6]. Put simply, this means that the splitting of bonds that include deuterium could be 18 times slower than bonds that include hydrogen. This massively greater-potential isotope effect of deuterium explains why so much of the possible toxicity studies have been carried out with deuterium.

The safety and possible biochemical or physiological toxicity have been addressed a number of times since the potential of such isotopes to be used in human nutritional research became apparent [7, 8]. Despite numerous studies in these areas, there can still be misunderstandings and misinformation in some situations that can inhibit or delay the use of these tracers in important nutritional research. Indeed, the titles of some of these papers, for example “Stable isotopes in clinical research: safety reaffirmed” [8], indicate a need to remind or inform researchers of the inherent safety of methods that use stable isotopes in clinical and public health research.

The aim of this mini review is therefore to consider that the safety of four key stable isotopes used in nutritional research reiterates and reaffirms their safety, yet again, almost 30 years after the publication of Jones and Leatherdale [8]. It is hoped that these data will be of use to new researchers in the field, as well as those considering the ethical or other implications of using these two stable isotopes in nutritional research.

## Deuterium

Deuterium is a stable isotope of hydrogen that was combined with oxygen to form deuterium oxide or heavy water in the early 1930s, very soon after its discovery [9]. Moreover, the potential use of deuterium as a stable isotope tracer in studies of human and animal metabolism was soon recognized. The first work involving deuterated linseed oil being fed to mice was reported in 1935. These pioneers, to their surprise, found that about a third of the labelled fatty acids were deposited in the adipose tissue of the mouse, as opposed to being liberated with water and carbon dioxide following oxidation [10].

Deuterium oxide has been produced in large quantities due to its role in nuclear reactors and nuclear energy research. In this role, deuterium oxide acts as a neutron moderator that reduces the speed of fast neutrons, and thus converts them into thermal neutrons that are required to initiate a nuclear chain reaction needed in this industry. This association with the nuclear industry has led to confusion in some cases, while deuterium oxide has been thought as radioactive when plainly it is not.

The potential physiological and/or biochemical toxicity of deuterium has been studied extensively over many years. Indeed, following its discovery in 1932, over 200 studies had been reported by the end of that decade, which dealt with the biological effects of deuterium. This intensity of research was partly due to the fact that the isotopes ^1^H and ^2^H have a significant relative difference in their masses, and therefore have the potential to have marked effects on metabolic and other biological systems [6]. Following on from these studies, it is generally accepted that side effects can be seen in animal models when total body water is enriched to between 10 and 15%, and death occurs at an enrichment of around 30–40%, although it should be remembered that such levels of enrichment will not be reached following a single dose in human studies [6]. Side effects included hypoglycaemia, hypothermia, alterations in ECG, muscle weakness, lymphopenia and hyperactivity [6].

One study that used deuterium to investigate cholesterol metabolism raised the enrichment in body water to between 0.05 and 0.12%, with no biological or physiological effects other than one case of dizziness [11]. It has been suggested that this was caused by changes in the specific gravity of the vestibular fluid in the inner ear.

Fortunately, and importantly, levels of enrichment that are associated with such side effects or death, are not required for the usual uses of deuterium to measure total body water, or in its other roles, such as in the doubly labelled water method for measuring total energy expenditure, or for assessing breast milk intake in infants. This was elegantly explained as far back as 1979, when one the most respected researchers in this area, WA Coward based in Cambridge, United Kingdom, responded to a letter in the Lancet seeking assurance that using deuterium in young infants was not a safety concern [12]. Coward reiterated that toxicity in humans occurs when at least 15% of total body water is replaced with deuterium oxide. He then pointed out that a single dose of deuterium oxide of 0.1 g/kg body weight (the dose that was being used in contemporary studies) would raise the deuterium concentration from a normal value of 150 p.p.m to 293 p.p.m, which is equivalent to 0.0293% of total body water, about 500 times less than the concentration required for toxic side effects, and up to 1300 times less than the levels associated with mortality.

## Oxygen (^18^O)

This isotope in the form of water (H_2_^18^O), can also be used to measure total body water by using exactly the same dilution methodology as that used with deuterium oxide. It has been suggested that ^18^O provides a more accurate measure of total body water when compared with using deuterium oxide as there is less non-aqueous exchangeable oxygen than hydrogen in the human body at physiological temperatures and pH. Thus, whilst a deuterium dilution space should be divided by 1.04 to adjust for this non-aqueous hydrogen, the ^18^O dilution space should be divided by only 1.01 to obtain a measure of total body water [13]. However, practically, this potentially greater accuracy has to offset against the fact that commercially available ^18^O is more expensive than deuterium.

The relatively small difference in mass between ^18^O and the most abundant isotope of oxygen, i.e. ^16^Oxygen, is one of the reasons that no adverse biological effects have been reported when ^18^O is used even in levels much higher than those needed for nutritional studies. Two often cited studies that support the fact that ^18^O appears to be harmless is the work of Spielman and Nau [14] and other data reported by Wolf et al. [15]. In the first of these studies, no side effects were noted in baboons, breathing air containing around 90% H_2_^18^O. In the second report also there were no side effects noted in mice who for three generations had been breathing air containing 90% ^18^O_2_ with the 3rd generation also consuming water as 90% H_2_^18^O.

In a calculation similar to that carried out by Coward [12] with deuterium, it has been stated that body water in humans contains about 1300 mg/kg body weight. Using H_2_^18^O to measure body water giving a standard dose of 60 mg ^18^O/kg body weight, lifts the enrichment of ^18^O in body water to about 1360 mg/kg body weight, an increase of <5% [16]. Also, if using ^18^O as part of the doubly labelled water technique to measure total energy expenditure, a dose of around 220 mg ^18^O/kg body weight proved successful when studying infants, but only raised the enrichment in the body to 1520 mg/kg, an increase of about 17% [17].

Both ^18^O and ^2^H, in the form of water, are often supplied with labels stating “unfit for human consumption” or “not for human use” or similar, which can cause alarm to ethics committees or other bodies. This is simply because these stable isotopes are not manufactured within the same specifications as compounds or substances designed to be a “food” as defined by many national regulatory bodies. The concern is that the manufacturers cannot guarantee that the product is free of bacteria and/or pyrogens. As suggested by Speakman [18] these concerns can be overcome by autoclaving the dose to be consumed in its bottle at around 65 °C to remove any possible bacterial presence. Others may prefer to use sterile 0.1-µm filters to remove bacteria and/or use ultrafiltration to remove any bacteria and possible pyrogens.

## Carbon (^13^C)

Carbon-13 has been used safely as a tracer in many studies over many years, for example, in the form of ^13^C-glucose, ^13^C-palmitate and ^13^C-leucine to investigate carbohydrate, fat and amino acid oxidation, respectively [6]. This isotope is also used in breath tests and examples include ^13^C-Urea to investigate the presence or absence of *Helicobacter pylori* in humans and as ^13^C-octanoate that can be used to assess rates of gastric emptying [19]. In typical tracer studies, the body pool of ^13^C is raised by only about 1% above background levels [7, 8] and often much less than this [6].

Studies of safety similar to those undertaken with ^18^O have been reported for ^13^C. The significant work of Gregg et al. is extensively cited as support for the safety in humans of this isotope. For example, almost 50 years ago Gregg et al. reported no adverse side effects with enrichment levels of ^13^C of up to 60% for 200 days in mice [20]. Also, in a paper the following year, Gregg also reported no teratogenity or embryotoxicity of ^13^C at enrichments of around 15–20% [21].

It is also noteworthy that the natural abundance of ^13^C is, when compared with other stable isotopes, quite high at about 1.1%. As a consequence the enrichment that occurs following a loading dose within a research protocol is usually small relative to the natural background level. These data plus the evidence from many other studies that have considered the safety of ^13^C “precludes any discernible risk of toxicity” according to Jones and Leatherdale [8].

## Nitrogen (^15^N)

There are fewer data available in the literature relating to the safety or side effects of using ^15^N in tracer studies. The isotope is commonly used to label amino acids in order to study the aspects of protein synthesis. Nevertheless, increases in the body pool are small in typical studies, with the value being quoted as changing from about 110 to about 150 mg/kg [6]. The capacity to increase the body pool safely with ^15^N has also been described as “virtually limitless” [7].

## Conclusion and summary

Countless studies in nutritional research have been completed safely and without incident over many years using stable isotopes, notably ^18^O, deuterium, ^13^C and ^15^N. No biological and/or physiological effects have been found when using ^18^O, ^13^C and ^15^N even at extremely high levels of enrichment far above those required in human nutritional tracer studies [14, 15]. Whilst adverse effects when using high enrichments of deuterium [6] have been described, once again the enrichment required to cause physiological changes are far in excess of those required for biological studies [12]. Both experienced and new potential users of the stable isotopes highlighted in this mini review should feel confident that significant information pertaining to body composition assessment and energy metabolism can be obtained safely when following established methods, and observing other standard safety precautions in their work.
