# Correction: Stable isotopes: their use and safety in human nutrition studies

**DOI:** 10.1038/s41430-020-00776-3

**Published:** 2020-10-15

**Authors:** Peter S. W. Davies

**Affiliations:** Child Health Research Centre Level 6, Centre for Children’s Health Centre, University of Queensland, 62 Graham Street, South Brisbane, QLD 4101 Australia

**Keywords:** Translational research, Obesity

Correction to: *European Journal of Clinical Nutrition*

10.1038/s41430-020-0580-0

This article was originally published under Nature Research’s License to Publish, but has now been made available under a CC BY 4.0 license. The PDF and HTML versions of the article have been modified accordingly.

